# C-reactive protein gene rs1205 polymorphism is associated with low-grade chronic inflammation in postmenopausal women

**DOI:** 10.1186/s40695-020-00051-2

**Published:** 2020-05-27

**Authors:** Iriane Prado de Santis, Juliana Dal-Ri Lindenau, Ramon Bossardi Ramos, Thais Rasia Silva, Gislaine Casanova, Karen Oppermann, Poli Mara Spritzer

**Affiliations:** 1 Gynecological Endocrinology Unit, Division of Endocrinology, Hospital de Clínicas de Porto Alegre, Rua Ramiro Barcelos, 2350, CEP, Porto Alegre, RS 90035 003 Brazil; 2 Department of Cell Biology, Embriology and Genetics, Universidade Federal de Santa Catarina, Florianópolis, SC Brazil; 3 Health Sciences School, Universidade Anhembi Morumbi, São Paulo, SP Brazil; 4 Division of Obstetrics and Gynecology, Hospital de Clínicas de Porto Alegre, Porto Alegre, RS Brazil; 5 Medical School of Universidade de Passo Fundo and São Vicente de Paulo Hospital, Rua Teixeira Soares 885/704, CEP, Passo Fundo, RS 99010-081 Brazil; 6 Department of Physiology, Laboratory of Molecular Endocrinology, Universidade Federal do Rio Grande do Sul, Porto Alegre, Brazil

**Keywords:** Menopause, Polymorphism, Inflammation, C-reactive protein, rs1205

## Abstract

**Background:**

Cardiovascular disease is the leading cause of death in postmenopausal women, and inflammation is a key mechanism involved in the pathogenesis of atherosclerosis. High-sensitivity C-reactive protein (hs-CRP) has been used as a biomarker of inflammation. Considering that *CRP* gene rs1205 polymorphism has been associated with hs-CRP circulating levels, we evaluated whether rs1205 genotypes influence the presence of low-grade chronic inflammation, acting as a marker of cardiovascular risk.

**Methods:**

We performed a cross-sectional study with biobanked blood samples from 327 postmenopausal women with no evidence of clinical disease. Genotyping for rs1205 C > T SNP of the *CRP* gene was done by real-time polymerase chain reaction with allelic discrimination assays.

**Results:**

Mean age was 55.6 ± 5.6 years. Mean body mass index (BMI) was 27.3 ± 4.7. Participants were divided according to hs-CRP levels: ≥3 mg/l (low-grade chronic inflammation) or < 3 mg/l. The frequency of allele C at rs1205 was 74.2% in the hs-CRP ≥ 3 mg/l group vs. 59% in the hs-CRP < 3 mg/l. In a multivariable model, higher prevalence of hs-CRP ≥ 3 mg/l was associated with CC genotype (PR 1.53; 95%CI 1.07–2.18; *p* = 0.018) and waist circumference ≥ 88 cm (PR 2.45; 95%CI 1.66–3.60; *p* < 0.001).

**Conclusions:**

*CRP* rs1205 CC homozygotes may be at higher risk of a low-grade chronic inflammatory status compared to individuals carrying the T allele.

## Introduction

Cardiovascular disease (CVD) is the leading cause of death in postmenopausal women. Some evidence suggests that CVD risk is increased in this population, perhaps as a result of declining ovarian estrogen secretion, aging, or both [1–3]. Menopause and ageing may also be associated with an unfavorable phenotype linked to changes in hormones such as estradiol, sex hormone binding globulin (SHBG), and follicle-stimulating hormone (FSH), as well as changes in cardiometabolic profile – including increases in blood pressure, triglycerides, and low-density lipoprotein cholesterol (LDL-c), and reduction in high-density lipoprotein cholesterol (HDL-c) [4–6]. Atherosclerosis, which underlies the occurrence of cardiovascular events, develops over decades, and low-grade chronic inflammation is a key mechanism in its initiation and progression [7]. Central adiposity, a marker of visceral adipose tissue, often increases in women during the menopause transition and post-menopause [8, 9]; emerging evidence suggests that inflammatory adipokines are released by this visceral fat depot, possibly contributing to increased cardiometabolic risk [10, 11].

C-reactive protein is a non-specific, acute phase reactant produced by the liver in response to pro-inflammatory stimuli. This protein is clinically used as a biomarker of inflammation, and changes in C-reactive protein levels may be associated with a variety of diseases [12, 13]. The development of high sensitivity laboratory assays for C-reactive protein (hs-CRP) has become an important tool to explore the role of this reactant as a predictor of cardiovascular events [14, 15]. hs-CRP assays exhibit good stability and reproducibility, as well as good sensitivity, with a low detection limit [16]. Many prospective studies have reported a positive association between hs-CRP levels and CVD, which seems to imply that the increase in hs-CRP levels reflects a gradual increase in cardiovascular risk [17–19].

Circulating hs-CRP levels have also been linked to genetic factors [20]. The heritability of C-reactive protein secretion has been estimated at 25 to 56% [21–23], suggesting that genetic variation is an important determinant of C-reactive protein levels. Several genes have been associated with C-reactive protein secretion, especially the *CRP* gene [24, 25]. The *CRP* gene encoding the C-reactive protein is located in chromosome region 1q23.2. A single nucleotide polymorphism (SNP), rs1205 C/T, located in the 3′ flanking region of the *CRP* gene, has been linked to circulating levels of C-reactive protein and may influence the expression of this gene [26]. rs1205 has been previously reported as a *CRP* 3′ UTR SNP, and its major C allele has been associated with higher C-reactive protein levels and increased risk of adverse cardiometabolic outcomes [27]. A National Health and Nutrition Examination Survey (NHANES) analysis with a population including 41% of postmenopausal women found an association between serum C-reactive protein concentration and rs1205 in adult US women [28]. An epidemiological study of men and women to determine aging risk factors (AGES-Reykjavik Study) showed that carriers of allele C of rs1205 captured the strongest effects on hs-CRP levels [29]..

The evidence that *CRP* gene SNPs are associated with CVD risk factors may provide further knowledge regarding disease susceptibility mechanisms. Studies with postmenopausal women are needed because of the increased central adiposity-related cardiovascular risk in this group. Therefore, the aim of the present study was to assess whether genotypes in the rs1205 SNP of the *CRP* gene influence the presence of low-grade chronic inflammation (hs-CRP ≥3 mg/l) considered a novel CV risk factor.

## Methods

### Study design and participants

This is a cross-sectional study of biobanked samples collected from a population of 327 post-menopausal women aged between 45 and 65 years without a history of clinical cardiovascular disease. These women were prospectively recruited and participated in studies conducted at our research center from 2005 to 2012 [27–29]. Briefly, the first study [30] was a randomized crossover trial comparing oral versus non-oral HT. Postmenopausal women consulting for climacteric symptoms at our outpatient clinic at the Hospital de Clínicas de Porto Alegre, Brazil, were consecutively enrolled if they met the following inclusion criteria: not having had a hysterectomy, last menstrual period between 6 months and 3 years before the beginning of the study plus FSH levels higher than 35 IU/L, not using any medication known to interfere with hormonal, glucose, or lipoprotein levels in the past 3 months, and not using steroidal or nonsteroidal anti-inflammatory drugs in the last 15 days. Patients with diabetes, endometrial thickness > 0.5 cm, clinically relevant abnormal mammogram, history of cancer, thromboembolism, or established cardiovascular disease were excluded. The present analysis used data obtained from participants after 3 months of oral or non-oral estradiol treatment.

The second study [31] was cross-sectional in design (third field visit) and nested in a longitudinal population-based study of menopausal status that aims to assess cardiovascular risk among pre-, peri-, and postmenopausal women through habitual physical activity, coronary artery calcium, abdominal fat and anthropometric measurements. The detailed study protocol has been described elsewhere [32]. Only data from postmenopausal women were included in the present study.

The third study [33] was also cross-sectional and focused on dietary protein intake and body composition. Inclusion criteria in the third study were age between 45 and 65 years, last menstrual period at least 1 year before the beginning of the study, FSH levels > 35 IU/L, and no use of hormone therapy (HT) in the past 3 months. Women with diabetes or previous diagnosis of heart disease and current smokers were excluded.

In the three studies, participants were interviewed using a standardized questionnaire covering demographic characteristics, clinical and gynecologic data, and use of HT for menopausal complaints. Physical examination did not reveal any limitations in mobility or cognition, and all participants appeared to be healthy. When responding to the standardized questionnaire, none of the participants reported previous renal or hepatic insufficiency, stroke, myocardial infarction, or thromboembolic events. In all three studies, written informed consent was obtained from all subjects at the time of recruitment for the clinical, laboratory, and genetic study components.

The serum samples that had been previously collected for hormonal assessment were stored in aliquots at − 80 °C. An additional blood sample collected on FTA Elute cards (GE Healthcare, Buckinghamshire, UK) was available for DNA extraction and polymorphism genotyping.

### Measurements

As previously reported [30, 31, 33], physical examination was performed with measurement of blood pressure, weight, height and waist circumference (WC). Body mass index (BMI) was calculated as weight in kg divided by the square of height in meters (kg/m^2^). Metabolic syndrome was defined by the presence of at least three of the following: triglycerides ≥150 mg/dl, HDL-c ≤ 50 mg/dl, glucose ≥100 mg/dl, systolic blood pressure ≥ 130 mmHg or diastolic blood pressure ≥ 85 mmHg and waist circumference ≥ 88 cm [34].

### Laboratory analyses

All samples were obtained between 08:00 AM and 10:00 AM. Blood samples were drawn after a 12-h overnight fast for determination of laboratory analyses. Total cholesterol, HDL-c, and triglycerides (TG) were determined by colorimetric-enzymatic methods (Bayer 1800 Advia System, Mannheim, Germany), with intra- and inter-assay coefficients of variation (CV) < 3%. Low-density lipoprotein cholesterol (LDL-c) was calculated using the Friedewald formula [35]. Glucose was quantitated by the hexokinase method (Advia 1800, Mannheim, Germany), with intra- and inter-assay CV < 3.4%. Serum insulin levels were measured using ECLIA (Roche Diagnostics, Mannheim, Germany), with sensitivity of 0.200 μIU/ml and intra- and inter-assay CVs of 2.0 and 4.3% respectively. The homeostasis model assessment of insulin resistance (HOMA-IR) index was calculated by multiplying insulin (μIU/ml) by glucose (mmol/l) and dividing this product by 22.5 [36]. Estradiol was measured by electrochemiluminescence (Roche Diagnostics, Mannheim, Germany) with sensitivity of 5.0 pg/ml with intra- and inter-assay CVs of 5.7 and 6.4%. Individual results below the limit of sensitivity of the test were considered as equal to 5.0 pg/mL for statistical analysis. Interleukin 6 (IL6) was measured in a subsample of 253 subjects, using multiplex immunoassay based on Luminex® xMAP (Merck KGaA, Darmstadt, Germany) with sensitivity of 0.2 pg/mL with intra- and inter-assay CVs of < 10 and < 15% respectively. High-sensitivity CRP [hs-CRP] was assayed with use of stored specimens, with a validated nephelometric method (Dade Behring Marburg, Marburg, Germany). Sensitivity was 0.17 mg/l, and intra-assay and inter-assay CV were 4.4 and 5.7% respectively. For data analysis, individual results below the limit of sensitivity were considered as equal to 0.17 mg/l. Values higher than 10 mg/l were excluded from the analysis due to possible interference of inflammatory processes. For the present analysis, participants were stratified into two groups according to hs-CRP as < 3 mg/l or ≥ 3 mg/l following National Academy of Clinical Biochemistry Laboratory Medicine Practice Guidelines. Levels < 1 mg/l indicate a low systemic inflammatory state and lower atherosclerotic risk; 1 to 3 mg/l indicates moderate vascular risk; and levels > 3 mg/l indicate high vascular risk when evaluated in the context of other risk factors [37, 38].

### Genotyping

Genomic DNA was extracted from the FTA Elute cards according to the manufacturer’s protocol (GE Healthcare). Genotyping for rs1205 C > T SNP of the *CRP* gene was done by real-time polymerase chain reaction with allelic discrimination assays (Taqman MGB Probes) following the manufacturer’s instructions (Applied Biosystems).

### Statistical analysis

Results are presented as means ± standard deviation (SD), medians and interquartile range, or percentages. Non-Gaussian variables were log-transformed for statistical analysis and reported after being back-transformed into their original units of measure. Student’s *t* test was used for comparisons between groups, and the chi-square (χ^2^) test was used to compare categorical variables. Yate’s correction was used when necessary. Genotypes were grouped according to the number of subjects observed in each category. T carriers were grouped for analysis (heterozygotes plus TT homozygotes vs. CC homozygotes). The relation of the outcome (CRP levels) with the variables analyzed was evaluated using prevalence ratios (PR) estimated by univariable Poisson regression with robust variance. The hs-CRP < 3 mg/l category was used as reference. Significant variables in the univariable model were inserted in a multivariable model. This method has been recommended as a more adequate alternative than logistic regression for cross-sectional studies with common binary outcomes [39, 40]. hs-CRP levels were also correlated with all the variables through Spearman correlation. Linear and multiple regression were performed to assess the relation between hs-CRP and the variables analyzed. All analyses were performed with SPSS for Windows v. 18.0 (SPSS Inc., Chicago, IL, USA). Data were considered to be significant at *p* < 0.05.

## Results

Table 1 summarizes the clinical and metabolic profile of the 327 postmenopausal women. The mean age of participants was 55.6 ± 5.6 years; 87% were white, and mean BMI was 27.3 ± 4.7.

**Table 1:** Clinical and metabolic features of 327 postmenopausal women

Variable	
Age, years	55.6 ± 5.6
Time since menopause, years	7 (2–10)
Body mass index, kg/m^2^	27.3 ± 4.7
Waist circumference, cm	87.1 ± 11.4
Systolic blood pressure, mmHg	126.0 ± 18.3
Diastolic blood pressure, mmHg	79.8 ± 11.6
Total cholesterol, mg/dl	209.1 ± 44.6
HDL-c, mg/dl	55.7 ± 12.9
LDL-c, mg/dl	126.3 ± 36.8
Triglycerides, mg/dl	118 (83–163)
Glucose, mg/dl	91 (85–97)
HOMA-IR	1.77 (1.25–2.65)
Insulin, μUI/ml	7.08 (5.4–9.7)
Metabolic syndrome, no (%)	229 (70.5%)
hs-CRP, mg/l	1.8 (0.12–9.9)
IL6, pg/mL	1.13 (0.78–1.77)
Estradiol, pg/ml	35.4 (11.2–70)
HT, n (%)	95 (29.1%)

Data are presented as means (± standard deviation) or as median (interquartile range), according to Gaussian or non-Gaussian distribution. Categorical variables (metabolic syndrome and HT) are presented as number of women (percentage)

*HDL-c* high-density lipoprotein cholesterol, *LDL-c* low density lipoprotein cholesterol, *HOMA-IR* homeostatic model assessment, *hs-CRP* high-sensitivity C-reactive protein, *IL6* interleukin 6, *HT* hormone therapy

The genotype frequencies were in accordance with Hardy-Weinberg equilibrium. rs1205 genotype frequency was 39.4% for the CC genotype, 47.4% for the heterozygous CT genotype, and 13.2% for the homozygous TT genotype. The allele frequencies were 0.63 for C and 0.37 for T, showing a similar pattern to these ones observed in European and American populations of 1000 genomes project data (www.internationalgenome.org).

Table 2 presents anthropometric, clinical and metabolic features according to plasma hs-CRP concentrations (< or ≥ 3 mg/l). The hs-CRP < 3 mg/l group had lower waist circumference, BMI, systolic blood pressure, triglycerides, insulin, and HOMA-IR, and higher HDL-c than the hs-CRP ≥3 mg/l group (*p* < 0.05). Metabolic syndrome was more prevalent (*p* = 0.040) and IL6 levels were higher in the hs-CRP ≥3 group (*p* < 0.001). CRP categories had similar proportions of self-reported White women, time since menopause, diastolic blood pressure, fasting glucose, total cholesterol, and LDL-c.

**Table 2:** Anthropometric, clinical and metabolic characteristics according to hs-CRP levels

	hs-CRP		
Variable	< 3 mg/l (*n* = 238)	≥3 mg/l (*n* = 89)	*p*-value
Age, years	55.2 ± 5.5	56.7 ± 5.6	**0.029**^**a**^
Time since menopause, years	4 (2–10)	7 (2–12)	0.157^a^
White participants, % (n)	88.6% (210)	85.1% (74)	0.503^b^
Waist circumference, cm (*n* = 292)^c^			
WC < 88 cm	66.35% (140)	35.80% (29)	**< 0.001**^**b**^
WC ≥ 88 cm	33.65% (71)	64.20% (52)	
Body mass index, kg/m^2^ (*n* = 287)^c^			
< 24.9	41% (85)	17.50% (14)	**< 0.001**^**b**^
25–30	44% (91)	41.25% (33)	
> 30	15% (31)	41.25% (33)	
SBP, mmHg	125.8 ± 17.4	131.2 ± 19.3	**0.021**^**a**^
DBP, mmHg	79.7 ± 11.1	82.2 ± 11.7	0.084^a^
Total cholesterol, mg/dl	214.6 ± 41.9	212.2 ± 48.1	0.662^a^
HDL-c, mg/dl	58.3 ± 14.4	54.4 ± 11.8	**0.030**^**a**^
LDL-c, mg/dl	130.4 ± 36.3	126.5 ± 36.7	0.410^a^
Triglycerides, mg/dl	115 (76–157)	131 (97–173)	**0.018**^**a**^
Glucose, mg/dl	94.1 ± 24.8	93.4 ± 18.3	0.946^a^
HOMA-IR	1.6 (1.2–2.4)	2.1 (1.5–3.1)	**0.026**^**a**^
Insulin, μUI/ml	7.4 (5.5–10.1)	9.1 (6.6–13.2)	**0.019**^**a**^
Metabolic syndrome, % (n)	26.2% (62)	38.6% (34)	**0.040**^**b**^
IL6, pg/mL (*n* = 279)^c^	1.02 (0.73–1.56)	1.43 (1.08–2.44)	**< 0.001**^**a**^
HT, % (n)	32% (76)	21% (19)	0.082^b^
Oral (*n* = 54)^c^	65.7% (44)	52.6% (10)	0.442^b^
Non-oral (*n* = 32)^c^	34.3% (23)	47.4% (9)	
T carriers, % (n)	65% (155)	48% (43)	**0.008**^**b**^

Values are expressed as mean (± standard deviation) or median (interquartile range) (25–75%) (^a^Student’s t test) or percentage (absolute number) (^b^χ^2^ test, with continuity correction when applicable). ^c^Number of subjects in sub-sample analyses. P-value of nonparametric variables was obtained after logarithmic transformation. Percentages of hs-CRP levels to categorical variables are shown in the rows. *SBP* systolic blood pressure, *DBP* diastolic blood pressure, *HDL-c* high-density lipoprotein cholesterol, *LDL-c* low-density lipoprotein cholesterol, *HOMA-IR* homeostatic model assessment. *HT* hormone therapy, *IL6* interleukin 6

The frequency of HT-users was slightly, but not significantly, higher among participants with hs-CRP < 3 mg/l (*p* = 0.082) (Table 2).

T carriers were more prevalent in the group with hs-CRP < 3 mg/l (65% vs. 48%, *p* = 0.008) (Table 2). hs-CRP plasma levels were significantly higher in CC homozygotes [2.1 (1.0–3.9)] compared to T carriers [1.5 (0.63–2.6), *p* = 0.004]. None of the other variables differed between the rs1205 genotypes (Table 3).

**Table 3:** Anthropometric, clinical and metabolic characteristics according to rs1205

	rs1205		
Variable	CC (*n* = 129)	T carriers (*n* = 198)	*p*-value
Age, years	55.4 ± 5.3	55.7 ± 5.7	0.596^a^
Time since menopause, years	5 (2–10)	5 (2–12)	0.978^a^
Waist circumference, cm			
WC < 88 cm	39.5% (73)	40.2% (53)	0.994^b^
WC ≥ 88 cm	60.5% (112)	59.8% (79)	
Body mass index, kg/m^2^ (*n* = 316)^c^			
< 24.9	31.2% (39)	36.1% (69)	0.633^b^
25–30	44.8% (56)	42.9% (82)	
> 30	24.0% (30)	20.9% (40)	
SBP, mmHg	125.9 ± 17.8	126.2 ± 18.7	0.902^a^
DBP, mmHg	80.2 ± 11.5	79.7 ± 11.7	0.714^a^
Total cholesterol, mg/dl	207.2 ± 46.1	210.5 ± 43.7	0.510^a^
HDL-c, mg/dl	55.7 ± 12.7	58.8 ± 14.6	0.300^a^
LDL-c, mg/dl	124.5 ± 40.2	127.5 ± 34.5	0.476^a^
Triglycerides, mg/dl	140.7 ± 141.8	139.6 ± 118.2	0.938^a^
Glucose, mg/dl	93.1 ± 18.3	94.2 ± 24.3	0.662^a^
HOMA-IR	1.7 (1.3–2.3)	1.8 (1.2–2.7)	0.229^a^
Insulin, μUI/ml	7.3 (5.7–9.8)	8.0 (5.6–11.6)	0.354^a^
Metabolic syndrome, % (n)	31.8% (41)	28.1% (55)	0.552^b^
hs-CRP, mg/l	2.1 (1.0–3.9)	1.5 (0.63–2.6)	**0.004**^**a**^
IL6, pg/mL (n = 279)^c^	1.13 (0.85–1.79)	1.13 (0.76–1.74)	0.285^a^
HT, % (n)	28.7% (37)	29.3% (58)	0.999^b^

Values are expressed as (± standard deviation) or median (interquartile range) (25–75%) (^a^Student’s t test) or percentage (absolute number) (^b^χ^2^ test, with continuity correction when applicable). ^c^Number of subjects in sub-sample analyses. *P*-value of nonparametric variables was obtained after logarithmic transformation. *SBP* systolic blood pressure, *DBP* diastolic blood pressure, *HDL-c* high-density lipoprotein cholesterol, *LDL-c* low-density lipoprotein cholesterol, *HOMA-IR* homeostatic model assessment, *hs-CRP* high-sensitivity C-reactive protein, *HT* hormone therapy, *IL6* interleukin 6

The allelic and genotypic distribution, according to the hs-CRP categories are presented in Table 4. Allele C at rs1205 had a frequency of 74.2% in the hs-CRP ≥ 3 mg/l group compared to a frequency for this allele of 59% in the hs-CRP < 3 mg/l group; the same was true for the genotype (CC 51.7%, CT 44.9% and TT 3.4%).

**Table 4:** Allelic and genotypic frequencies according to categories of hs-CRP

	hs-CRP < 3 mg/l	hs-CRP ≥ 3 mg/l	***p***-value
C	281 (59.0%)	132 (74.2%)	**0.001**
T	195 (41.0%)	46 (25.8%)	
CC	83 (34.9%)	46 (51.7%)	**0.001**
CT	115 (48.3%)	40 (44.9%)	
TT	40 (16.8%)	3 (3.4%)	

*hs-CRP* High-sensitivity C-reactive protein

Prevalence ratios for hs-CRP ≥3 mg/l according to genotype, metabolic syndrome, age, IL6 level, HOMA-IR, SBP, HDL-c, triglycerides, insulin, waist circumference and HT are shown in Table 5 (Model 1). A higher prevalence of hs-CRP ≥3 mg/l was associated with *CRP* rs1205 CC (PR 1.64; 95%CI 1.15–2.33; *p* = 0.006), metabolic syndrome (PR 1.50; 95%CI 1.05–2.15; *p* = 0.025), age (PR 1.03; 95%CI 1.01–1.06; *p* = 0.024), IL6 level (PR 1.03; 95%CI 1.01–1.05; *p* = 0.003), SBP (PR 1.01; 95%CI 1.002–1.02; *p* = 0.013), HDL-c (PR 0.98; 95%CI 0.97–0.99; *p* = 0.024) and waist circumference ≥ 88 cm (PR 2.46; 95%CI 1.67–3.64; *p* < 0.001). Based on the results obtained with univariable models, multivariable models were tested. The final multivariable model (Model 2 in Table 5) included having the CC genotype (PR 1.53; 95%CI 1.07–2.18; *p* = 0.018) and waist circumference ≥ 88 cm (PR 2.45; 95%CI 1.66–3.60; *p* < 0.001). The remaining variables were not significant in the multivariable model.

**Table 5:** Association analysis of prevalence ratios for clinical and metabolic variables and hs-CRP ≥3 mg/l

	Model 1		Model 2	
Variable	PR (95% CI)	*p*-value	PR (95% CI)	*p*-value
rs 1205				
CC	1.64 (1.15–2.33)	**0.006**	1.53 (1.07–2.18)	**0.018**
T carriers	1		1	
MS				
Yes	1.50 (1.05–2.15)	**0.025**	1.19 (0.79–1.80)	0.404
No	1		1	
Age, years	1.03 (1.01–1.06)	**0.024**	1.02 (0.99–1.06)	0.224
IL6, pg/mL	1.03 (1.01–1.05)	**0.003**	1.02 (0.99–1.04)	0.052
HOMA-IR	1.07 (0.99–1.16)	0.085		
SBP, mmHg	1.01 (1.002–1.02)	**0.013**	1.00 (0.99–1.02)	0.967
HDL-c, mg/dl	0.98 (0.97–0.99)	**0.024**	1.00 (0.98–1.02)	0.943
Triglycerides, mg/dl	1.001 (1.000–1.002)	0.052		
Insulin, μUI/ml	1.01 (0.99–1.03)	0.122		
HT				
Yes	0.66 (0.42–1.04)	0.072		
No	1			
WC				
< 88 cm	1		1	
≥ 88 cm	2.46 (1.67–3 64)	**< 0.001**	2.45 (1.66–3.60)	**< 0.001**

*hs-CRP* high-sensitivity C-reactive protein, *SBP* systolic blood pressure, *HT* hormone therapy, *MS* metabolic syndrome, *WC* waist circumference, *HOMA-IR* homeostatic model assessment, *IL6* interleukin 6, *PR* prevalence ratio, *CI* confidence interval

Model 1: PR for each variable

Model 2: multivariable Poisson regression including all the variables that were significant in the univariable analysis. Only the polymorphism and waist circumference remained significant in the final model

Similar correlations were obtained when hs-CRP was analyzed as a continuous variable. Age (B = 0.018, 95%CI 0.009–0.028; *p* < 0.001), waist circumference (B = 0.015; 95%CI 0.011, 0.019; *p* < 0.001), SBP (B = 0.006; 95%CI 0.003, 0.009; *p* < 0.001), IL6 level (B = 0.017; 95%CI 0.004, 0.030; *p* = 0.010) and CC genotype (B = 0.154; 95%CI 0.048, 0.260; *p* = 0.005) were significantly associated with hs-CRP in the univariable linear regression. In the multivariable regression, age, waist circumference and genotype remained significantly associated with hs-CRP.

## Discussion

In the present study, a higher frequency of the CC genotype (vs. T carriers) of *CRP* SNP rs1205 was observed in postmenopausal women presenting with CRP levels ≥3 mg/l. In fact, the number of women with hs-CRP levels ≥3 mg/l is expected to be about 50% higher in the CC genotype group as compared to T carriers. While the association of *CRP* SNP rs1205 with metabolic alterations and cardiovascular risk factors has been observed in different populations [20, 25, 41–46], to the best of our knowledge this is the first study to show that association in a specific sample of postmenopausal women without history of clinical disease.

Studies regarding *CRP* gene polymorphisms and risk of CVD outcomes are scarce and conflicting. A prospective population-based study that included men and women aged 55 years and older found no association between SNP rs1205 or haplotypes and coronary heart disease [47]. In turn, another study with predominantly Caucasian men and women observed that the C alleles of rs1205 and rs18000947 were related to elevated risk of coronary heart disease [48]. In addition, the Cardiovascular Health Study, which enrolled 3941 men and women aged 65 years or older, showed that the T allele of rs1205 was associated with lower hs-CRP levels and decreased CVD mortality [45].

Indeed, haplotype studies are usually more robust in evidencing the effects of gene polymorphisms on clinical outcomes. However, rs1205 seems to be the SNP that captures the strongest effects of the *CRP* gene on C-reactive protein levels [23]; the other SNPs that have been included in the haplotypes are in complete linkage disequilibrium with rs1205, and do not capture any signal beyond that detected between rs1205 and hs-CRP levels [45].

Circulating hs-CRP concentration is well-recognized as a non-specific marker of low-grade chronic inflammation [49], and is influenced by age, obesity, sex, smoking status, use of medications, and genetic factors [50]. Moreover, prospective studies have shown a strong and consistent association between elevated hs-CRP concentrations and various CVD endpoints [51, 52]. Some studies have also reported an association between ethnicity and higher CRP levels or subclinical CVD in black women [53, 54]. In the present study, the proportion of white women was high, and similar in the two CRP categories. In the South of Brazil, about 83% of the population are white and descend from Italian and German immigrants. The remaining 17% correspond to multiracial and black people (https://sidra.ibge.gov.br/Tabela/3175#resultado). A similar ethnicity pattern was observed in our study, and the small number of non-white postmenopausal women in our sample precludes any definitive conclusions.

We found that the anthropometric and metabolic profile of women with hs-CRP levels ≥3 mg/l was worse than that of women with hs-CRP levels < 3 mg/l. Interestingly, the prevalence of metabolic syndrome and IL6, a more robust marker of low-grade, chronic inflammation was also higher in the presence of hs-CRP levels ≥3 mg/l. These findings are in accordance with previous studies [10, 55–57] and suggest an association between the metabolic syndrome and a chronic inflammatory response expressed by increased cytokine secretion.

In addition, our participants with hs-CRP ≥3 mg/l had higher BMI, waist circumference, and HOMA-IR. In this sense, increased abdominal fatty tissue in postmenopausal women represents an important source of cytokines – which stimulate hs-CRP production [9, 10], especially IL6, enhanced by the interaction with IL1 in hepatocytes. In fact, the hypoxia caused by adipocyte hypertrophy stimulates the activation of intracellular nuclear factor-κB (NF-κB), which regulates immune response and induces production of tumor necrosis factor-α (TNF-α) and interleukins (IL). IL6 is thought to be the principal cytokine involved in hs-CRP release, and circulating levels derive mostly from adipose tissue [58]. This mechanism might explain the increased levels of hs-CRP and inflammatory cytokines, such as IL6 and TNF-α, in obese individuals, and also the positive correlation between these increases and BMI or waist circumference.

CVD risk increases in women at midlife [3], a period coincident with the menopausal transition [59]. If administered to women with climacteric symptoms at the first years after menses cessation, HT has been shown to improve cardiovascular risk factors [60]. We did not, however, observe any difference in this variable related to hs-CRP categories. This finding seems to underscore the complexity of HT administration route and dose – linked on the one hand to the first pass liver metabolism for oral route estrogen therapy, and on the other hand to the estrogen-receptor binding affinity and capacity of different types of estrogen and doses [61]. Indeed, full-dose oral estrogen therapy has been associated with an increase in plasma inflammatory markers, such as hs-CRP [62]. In turn, low doses [5] and the non-oral route have not been associated with changes in hs-CRP levels [30, 63].

One limitation of this study is the relatively small sample size of 327 participants for analysis of genotype/phenotype associations, which does not allow supplemental evaluations. However, the effect sizes observed in our sample are similar to those reported in other populations. Furthermore, consistent results were obtained with analysis of hs-CRP as either a categorical or continuous variable. Another limitation is the use of combined data obtained from a population-based study but also from convenience samples included in a clinical trial and an observational study. Therefore, the present results might not be generalizable. Conversely, a strength of our study is the focus on a less well represented ethnic group, postmenopausal women from southern Brazil.

## Conclusion

In conclusion, the present data from a sample of postmenopausal women with no evidence of clinical disease suggest that *CRP* rs1205 CC homozygotes may be at higher risk of a low-grade chronic inflammatory status compared to individuals carrying the T allele. Further longitudinal studies with other menopausal populations are needed in order to gauge the relationship of SNP rs1205 with cardiovascular events and the relevance of ethnic differences for these outcomes.

## Data Availability

All data generated or analyzed during this study are included in this manuscript and in previous publications: Colpani V, et al. Menopause. 2013;20(5):525–31. doi: 10.1097/GME.0b013e318271b388; Casanova G, et al. Climacteric. 2015;18(1):86–93. doi: 10.3109/13697137.2014.940309; Silva TR, Spritzer PM. Menopause. 2017;24(5):502–509. doi: 10.1097/GME.0000000000000793.

## Abbreviations

BMI: Body mass index
CV: Coefficient of variation
CVD: Cardiovascular disease
FSH: Follicle-stimulating hormone
HDL-c: High density lipoprotein cholesterol
hs-CRP: High-sensitivity C-reactive protein
HOMA-IR: Homeostatic model assessment
HT: Hormone therapy
IL-1: Interleukin 1
IL-6: Interleukin 6
LDL-c: Low density lipoprotein cholesterol
NF-κB: Nuclear factor-κB
PR: Prevalence ratio
SHBG: Sex hormone binding globulin
SNP: Single nucleotide polymorphism
TG: Triglycerides
TNF-α: Tumor necrosis factor-α
WC: Waist circumference
